# Tuberculosis1Presented at the annual meeting of the Veterinary Association of Manitoba.

**Published:** 1900-08

**Authors:** W. H. Lake

**Affiliations:** Miami, Manitoba


### TUBERCULOSIS.[1]

By W. H. Lake, V.S.,
MIAMI, MANITOBA.

Tuberculosis, commonly known as consumption, is a fatal, contagious, and infectious disease, very destructive to human and animal life, and which has baffled the skill of the medical profession to find a cure for. It occurs in man and all the domesticated animals. Cattle, swine, and poultry are most frequently affected; horses next; sheep and goats next; dogs and cats still less.

*Definition.* Tuberculosis is a contagious disease characterized by the formation of tubercules or nodules, which vary in size from a pinhead to that of a walnut, and contain the bacillus tuberculosis, which produces the disease.

*Etiology.* The disease cannot develop without the germ, therefore the germ has to find its way into some part of the system before the disease can be produced, and the most prolific way is from expectorated bacilli which are lying on the ground or floors, become dried, are ground up, and the germs floating in the air are inhaled. In a weak or debilitated system they are liable to find a culture ground, from which the germs grow and the disease is developed. The germs are also sometimes found in the feces and milk of the cow. Drinking the milk has produced the disease in children, calves, and pigs. The bacillus of one mammal multiplies in the bodies of other mammals. Dogs and poultry have been infected by expectorated bacilli from human patients having consumption.

*Semeiology.* Generally the first symptom noticed is a husky cough. Auscultation reveals a dull, semi-solid sound. Generally in the early stage there is little or no constitutional disturbance; but when the system begins to suffer from lack of nutrition the appetite may be completely lost, emaciation takes place, tuberculous patches may appear on sides, hydrothorax may occur as a complication, general debility and death, due to an exhausting diarrhœa.

*Pathology.* The first step to be taken is the discovery of the germ which, having been taken in with the food into the stomach, passes through the walls of the intestines, rarely affecting those organs; then passing into the circulation and

[1] Presented at the annual meeting of the Veterinary Association of Manitoba.

lodging in some part of the body. Hamilton says they go in colonies of three, five, or nine, but usually five, and having found a suitable place to locate in they begin reproducing themselves. The result is inflammation, with degeneration of a caseous nature. This degeneration may be fibrous or hyaline. This spot is the tubercle, and is generally about the size of a millet-seed, and as more tissue is involved it grows and spreads. Then exudation and formation of neoplastic tissue as a result, the tissue being nearly always fibrous at first and the germ not too active. In the tubercle is a deposit of calcareous salts which incloses the tubercle in a fibrous capsule and renders it harmless for all time to come. This is the course of a mild case which ends favorably. This is the reason why so many germs which are inhaled do not cause tuberculosis; but when the bacilli find a suitable culture ground they increase in numbers very fast, and one colony after another is started. As a consequence the tubercles spread rapidly until as many as a dozen places may be affected before death takes place.

In some cases where the bacilli work with great activity we get inflammation, followed by rapid ulceration, decay, and death of part or parts affected; lung tissue is destroyed, blood vessels are eaten in two, and we have what has been termed the hemorrhage of consumption. Cases have been seen where the contents of sacks or capsules were liquid. This is due to not being inclosed in calcareous matter.

Tuberculosis is divided into several stages: First, caseous without inflammation; second, exudation and formation of a fibrous capsule; third, ulceration; fourth is where lime-salts are deposited around the caseous matter and the lung looks as if it were covered with lumps of chalk, putrefactive matter is absorbed and, passing through the blood, causes blood-poisoning.

*Diagnosis in autopsy.* This I consider should be of great interest to the veterinary profession at large, as by it we would be able to tell whether an animal had tuberculosis or not, providing we had never seen the animal before. The diagnostic features are six or seven: 1. The presence of miliary tubercles or nodules, either running in lines. 2. Arranged in groups or distributed evenly through the affected organ. 3. The nodules being about the size of a millet-seed and of uniformity of size. 4. Are hard and of a cartilaginous nature. 5. Can be easily dissected from the tissue. 6. Color usually gray, but may be of a creamy color.

*Parts liable to infection.* The lungs are the most likely organ to be affected, as the germs are taken in by respiration, then the spleen, kidneys, liver, brain, pleura, peritoneum, and germs have been found in the iris of the eye and aqueous humor; bones of human beings are often affected, and this has been termed scrofula.

*Treatment.* Where deemed advisable to resort to treatment the animal should have good nourishing food—oily foods—as linseed-meal, oil-cake, and in human beings cod-liver oil, fat meats, good nursing, and good healthy surroundings.

*Prophylactic treatment.* Undoubtedly the disease is contagious, and where a case is met with which looks suspicious it should be subjected to the tuberculin-test, and if it reacts should at once be destroyed, as the danger is great both to human and animal life in keeping such an animal around, especially a cow. If you inspect meats and find any ulceration from this disease, condemn it as unfit for use, and always beware of the meat or milk from a coughing cow. I think the tuberculin-test is so well understood by qualified veterinarians as to need no explanation here.

